# Antiplatelet Antibodies Do Not Predict the Response to Intravenous Immunoglobulins during Immune Thrombocytopenia

**DOI:** 10.3390/jcm9061998

**Published:** 2020-06-25

**Authors:** Thomas Rogier, Maxime Samson, Guillaume Mourey, Nicolas Falvo, Nadine Magy-Bertrand, Sethi Ouandji, Jean-Baptiste Picque, Hélène Greigert, Christelle Mausservey, Arthur Imbach, Thibault Ghesquière, Laurent Voillat, Denis Caillot, Eric Deconinck, Bernard Bonnotte, Sylvain Audia

**Affiliations:** 1 Service de Médecine Interne et Immunologie Clinique, Centre de Référence Constitutif des Cytopénies Auto-immunes de l’adulte, Centre Hospitalo-Universitaire Dijon Bourgogne, Université de Bourgogne Franche Comté, 21000 Dijon, France; thomas.rogier@chu-dijon.fr (T.R.); maxime.samson@u-bourgogne.fr (M.S.); nicolas.falvo@chu-dijon.fr (N.F.); sethi.ouandji@chu-dijon.fr (S.O.); helene.greigert@chu-dijon.fr (H.G.); thibault.ghesquiere@chu-dijon.fr (T.G.); bernard.bonnotte@chu-dijon.fr (B.B.); 2 Laboratoire d’Hématologie et d’Immunologie Régional, Établissement Français du Sang (EFS) Bourgogne/Franche-Comté, 25000 Besançon, France; guillaume.mourey@efs.sante.fr (G.M.); arthur.imbach@efs.sante.fr (A.I.); 3 Service de Médecine Interne, Centre Hospitalo-Universitaire, Université de Bourgogne Franche-Comté, 25000 Besançon, France; nmagy@chu-besancon.fr; 4 Service de Médecine Interne, Centre Hospitalier, 89000 Auxerre, France; jbpicque@ch-auxerre.fr; 5 Service de Médecine Interne, Centre Hospitalier William-Morey, 71100 Chalon/Saône, France; christelle.mausservey@ch-chalon71.fr; 6 Service d’Hématologie et Oncologie, Centre Hospitalier William-Morey, 71100 Chalon/Saône, France; laurent.voillat@ch-chalon71.fr; 7 Service d’Hématologie, Centre Hospitalo-Universitaire Dijon Bourgogne, 21000 Dijon, France; denis.caillot@chu-dijon.fr; 8 Service d’Hématologie, Centre Hospitalo-Universitaire, Université de Bourgogne Franche-Comté, 25000 Besançon, France; edeconinck@chu-besancon.fr

**Keywords:** immune thrombocytopenia, antiplatelet antibodies, IVIg

## Abstract

Immune thrombocytopenia (ITP) is a rare autoimmune disease due to autoantibodies targeting platelet glycoproteins (GP). The mechanism of platelet destruction could differ depending on the specificity of antiplatelet antibodies: anti-GPIIb/IIIa antibodies lead to phagocytosis by splenic macrophages, in a Fcγ receptor (FcγR)-dependent manner while anti-GPIb/IX antibodies induce platelet desialylation leading to their destruction by hepatocytes after binding to the Ashwell–Morell receptor, in a FcγR-independent manner. Considering the FcγR-dependent mechanism of action of intravenous immunoglobulins (IVIg), we assumed that the response to IVIg could be less efficient in the presence of anti-GPIb/IX antibodies. We conducted a multicentric, retrospective study including all adult ITP patients treated with IVIg who had antiplatelet antibodies detected between January 2013 and October 2017. Among the 609 identified, 69 patients were included: 17 had anti-GPIb/IX antibodies and 33 had anti-GPIIb/IIIa antibodies. The response to IVIg was not different between the patients with or without anti-GPIb/IX (88.2% vs. 73.1%). The response to IVIg was better in the case of newly diagnosed ITP (odds ratio (OR) = 5.4 (1.2–24.7)) and in presence of anti-GPIIb/IIIa (OR = 4.82 (1.08–21.5)), while secondary ITP had a poor response (OR = 0.1 (0.02–0.64)). In clinical practice, the determination of antiplatelet antibodies is therefore of little value to predict the response to IVIg.

## 1. Introduction

Immune thrombocytopenia (ITP) is an autoimmune disease partly mediated by antibodies that target platelet glycoproteins (GP) such as GPIIb/IIIa (fibrinogen receptor), GPIb/IX (von Willebrand factor receptor) and GPIa/IIa (collagen receptor). Until recently, the destruction of platelets following their opsonization by autoantibodies was thought to be solely mediated by splenic macrophages [1,2]. The regulation of platelet clearance and production is now better understood. Senescent platelets undergo desialylation leading to their binding to the Ashwell–Morell receptor (AMR) expressed on hepatocytes, responsible for their removal from circulation [3]. Interestingly, it has been shown in ITP that anti-GPIb/IX antibodies are responsible for platelet desialylation, conversely to anti-GPIIb/IIIa antibodies [4]. Hence, in the presence of anti-GPIIb/IIIa antibodies, the destruction of platelets is mediated by splenic macrophages and depends on Fcγ receptor (FcγR) recognition, whereas anti-GPIb/IX antibodies cause platelet desialylation leading to their destruction in the liver [4].

The determination of antiplatelet antibodies is now routinely performed by the direct monoclonal antibody immobilization of platelet antigens assay (MAIPA), with an overall sensitivity of 53% and a specificity of 93% [5]. However, the value of antiplatelet antibodies in clinical practice is partly known. Anti-GPIIb/IIIa antibodies have been associated with a chronic outcome and a higher risk of bleeding [6]. Moreover, the response to steroids [7] or intravenous immunoglobulins (IVIg) [8,9] seems to be better in the presence of anti-GPIIb/IIIa antibodies compared to anti-GPIb/IX. In this context, a higher response rate is achieved with rituximab in the presence of anti-GPIIb/IIIa antibodies [10].

IVIg are used in ITP as a rescue therapy when severe bleeding symptoms are present [11,12], with an overall response rate of 72–81% [12]. Although the mechanisms of action of IVIg are not completely understood, part of their immunomodulatory properties depends on the Fc portion of immunoglobulin G (IgG) and the inhibition of human mononuclear cell phagocytosis [13]. Whether the response to IVIg depends on the specificity of antiplatelet antibodies in ITP had not been extensively assessed. In a mouse model of ITP, IVIg prevent the ITP induced by anti-GPIIb/IIIa while they were inefficient in the case of anti-GPIb/IX [14]. This raised the possibility that IVIg could be less efficient in ITP patients harboring anti-GPIb/IX, which was strengthened by the data of a cohort of 17 patients showing a response to IVIg in 7/7 cases in the absence of anti-GPIb/IX antibodies, but only 3/10 patients who had anti-GPIb/IX antibodies [9]. The largest study included 156 treated with IVIg and reported a response in 36.4% of patients with anti-GPIb/IX compared to 80% of patients without anti-GPIb/IX [8]. However, none of the patients received steroids concomitantly with IVIg, which is usually recommended to achieve a better response [15].

Thus, we aimed to address in our cohort whether anti-GPIb/IX antibodies are associated with a lower response to IVIg in clinical practice, and to determine the predictive factors of response to IVIg.

## 2. Materials and Methods

A retrospective and multicentric study was conducted in Bourgogne-Franche/Comté, France, including patients older than 16 and diagnosed with ITP within one of the tertiary hospitals (University hospital of Dijon or Besançon, hospital of Auxerre or Chalon/Saône). The study was conducted in accordance with the Declaration of Helsinki and was approved by the institutional review board of the University Hospital of Dijon and the local ethics committee (Comité de Protection des Personnes Est I), who waived the requirement for informed consent. Patients who had antiplatelet antibodies measured between the 1st January 2013 and the 31st October 2017 and who were treated with IVIg were included.

ITP was defined as a platelet count < 100 G/L after the exclusion of other diagnoses, and depending on disease duration, was referred to as newly diagnosed (0–3 months), persistent (>3–12 months) or chronic (>12 months), as recommended in international guidelines [12].

Antiplatelet antibodies were screened by flow cytometry. In the case of positivity, defined by a mean fluorescence intensity above 25 units, a direct monoclonal antibody immobilization of platelet antigens (MAIPA) [16] was performed to characterize the target of the antiplatelet antibody. Platelet antibodies were measured by the French national blood service (Etablissement Français du Sang, EFS) of Besançon.

The need for IVIg treatment was determined by the clinician taking care of the patient. Depending on the clinician’s experience and the clinical characteristics of the patients, three different regimens of IVIg were used in accordance with international guidelines [12]: (1) 1 g/kg at day 1 and 2, (2) 1 g/kg at day 1, repeated at day 3 when the platelets were < 30 G/L, or (3) 0.4 g/kg/day for a maximum of 5 days, with a treatment interruption when the platelet count was > 30 G/L. Response to IVIg was defined as a platelet count > 30 G/L during the first week after IVIg initiation, with at least a doubling of the basal count. In accordance with the French guidelines, steroids were often associated with IVIg (prednisone, 1 mg/kg/day for 3 weeks).

Quantitative values were reported as median (1st–3rd interquartile) and the qualitative data as percentages. Categorical variables were compared with chi-2 or Fisher’s exact tests, and continuous variables were compared with the Student’s t-test or Mann–Whitney test, as appropriate. A logistic regression was performed to identify the factors associated with the response to IVIg. Candidate variables were selected among them with *p*-value < 0.25 in the univariate analysis. Results are expressed as an odds ratio (OR) with 95% confident interval (CI 95%). Statistical significance was defined as a *p*-value < 0.05 (two-tailed). Statistical analyses were performed with Epi Info software.

## 3. Results 

### 3.1. Patient Characteristics

**Figure 1:**
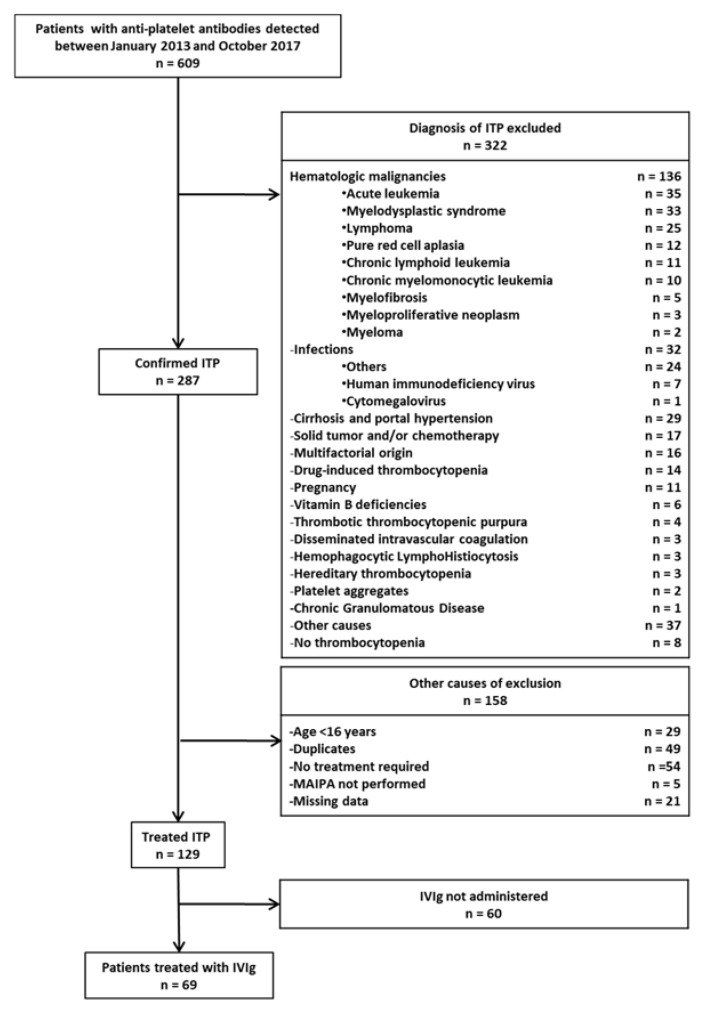
Flowchart of the study. ITP = immune thrombocytopenia; IVIg = Intravenous Immunoglobulin; MAIPA = monoclonal antibody immobilization of platelet antigens assay.

Among the 609 medical records screened, 69 patients were included after the exclusion of patients with other diagnoses than ITP or ITP patients that were not treated with IVIg or who did not have an antiplatelet antibody measurement (Figure 1).

Anti-GPIb/IX antibodies were detected in 17 patients (anti-GPIb/IX^+^), among which only one patient had no anti-GPIIb/IIIa associated. Anti-GPIb/IX were not detected in 54 patients (anti-GPIb/IX^−^). Clinical characteristics were not different between the patients with or without anti-GPIb/IX (Table 1). Median age at IVIg administration was 62.7 (41.8–76.4) years, with a female/male sex ratio of 1.16 and a median platelet count of 7 (5–18) G/L. The course of ITP was mainly newly diagnosed (62.3%), followed by chronic ITP (33%) and persistent ITP (4.3%). ITP was considered secondary in 21 patients with hematologic malignancies (*n* = 9), systemic lupus (*n* = 6), viral infections (*n* = 4), Behçet disease and unclassified auto-immune disease (*n* = 1 each).

### 3.2. Response to IVIg According to the Presence of Anti-GPIb/IX Antibodies

As depicted in Table 2, the overall response to IVIg was 76.8%. Anti-GPIb/IX were the unique antiplatelet antibodies identified in only one patient who did not respond to IVIg. Anti-GPIIb/IIIa in the absence of anti-GPIb/IX were detected in 24.6% of the cohort, with a response rate of 82.4%. Both antibodies were present in 23.2% with a response rate of 93.8%, while they were undetectable in most patients (50.7%) with a response rate of 68.6%.

In univariate analysis, the presence of anti-GPIb/IX was not associated with the response rate to IVIg (88.2% vs. 73.1%, *p* = 0.32).

Sub-group analyses were performed considering different the age groups at diagnosis or IVIg administration, patient sex, the course and the nature (primary or secondary) of ITP, and the antiplatelet antibody specificities. As depicted in Table 3, no difference was observed between the patients with and without anti-GPIb/IX. Of note, the concomitant use of steroids with IVIg did not modify the response to IVIg (88.9% without vs. 75% with steroids; OR = 0.38 (0.01–3.25), *p* = 0.67).

### 3.3. Predictive Factors of Response to IVIg

Univariate analyses of the factors associated with the response to IVIg are presented in Table 4. Patients with anti-GPIIb/IIIa had a better response to IVIg. Response to IVIg tended to be better in newly diagnosed ITP patients compared to chronic ITP whereas sex, age at diagnosis or treatment initiation, and the primary or secondary nature of ITP had no impact on the IVIg efficacy.

In multivariate analysis, newly diagnosed ITP and anti-GPIIb/IIIa antibodies were the two variables independently associated with a better response to IVIg (OR = 5.4 (1.2–24.7), *p* = 0.03 and OR = 4.82 (1.08–21.5), *p* = 0.04, respectively). By contrast, the response to IVIg was lower in the case of secondary ITP (OR = 0.1 (0.02–0.64), *p* = 0.01).

### 3.4. Response to Splenectomy According to the Presence of Anti-GPIb/IX Antibodies

As the destruction of platelets is thought to occur in the liver in the presence of anti-GPIb/IX, the response to splenectomy depending on platelet antibodies was also investigated. The overall response to splenectomy was 63.4% (7/11 patients) and was similar whatever the antiplatelet antibody profile (Table 5).

## 4. Discussion

The characteristics of our patients are in line with the general population of ITP with a median age of 60.3 years which is in accordance with the incidence peak observed after 60 [17]. IVIg were most often started during the first 3 months of diagnosis, as newly diagnosed ITP patients represented 62.3% of the cohort. The frequency of secondary ITP was overrepresented, concerning 30.4% of our patients, compared to 21% of patients above 50 years as observed in France [17].

The proportion of patients without antiplatelet antibodies detected by MAIPA was similar to previous papers that reported a frequency around 50% [18,19,20,21], excepted for the last studies that did not detect antiplatelet antibodies in only 20% [22,23]. Unsurprisingly, anti-GPIIb/IIIa antibodies were the most frequently detected, either isolated (24.6%) or associated with anti-GPIb/IX (23.2%), which is consistent with previous studies (10.7–23.5% and 17.5–61.4%, respectively) [18,19,20,21,22,23]. However, the proportion of patients with isolated anti-GPIb/IX was particularly low in our study (1.4%), whereas these antibodies usually account for 3.1–13.3% [18,19,20,21,22,23]. The reason for such a discrepancy was not clear but could be due to a bias of selection as antiplatelet antibodies were not systematically and prospectively measured in our cohort, in accordance with the guidelines [12]. Moreover, we acknowledge that the strategy for the detection of antiplatelet antibodies did not completely fulfill the guidelines, as it is not recommended to use flow cytometry to detect antiplatelet antibodies due to its low specificity [24]. However, it is unlikely that the use of flow cytometry as a first step for the detection of antibodies has led to the non-detection of antiplatelet antibodies as its sensitivity is of 90% [25].

The fact that isolated anti-GPIb/IX were detected in only one patient had probably participated to not confirm the results of the largest study conducted to date in 156 ITP patients, which reported a response to IVIg in 31.3% patients with isolated anti-GPIb/IX and in 76.6% with isolated anti-GPIIb/IIIa [8]. In contrast, we observed a better response in the patients who had both anti-GPIb/IX and anti-GPIIb/IIIa as reported in this study (93.8% vs. 41.2%) [8]. Although the small number of patients with GPIb/IX antibodies that received IVIg is a limiting factor to draw strong conclusions, our results are consistent with a previous study that showed no correlation between the presence of anti-GPIb/IX and the response to IVIg in 59 patients who had anti-GPIb/IX antibodies from a cohort of 260 patients [23]. This underlines the complex interplay of mechanisms of platelet destruction at a patient level and suggests that antiplatelet antibodies are not a proper surrogate marker to predict the response to IVIg. In fact, it could be more appropriate to assess the level of desialylation of platelets, as the effect of anti-GPIb/IX antibodies on platelets depends on the epitope they bind on GPIbα and do not always lead to their desialylation [26]. Along this line, it has recently been reported that platelet desialylation was associated with non-response to first line therapies in ITP [27].

We acknowledge that the use of steroids could be a confounding factor and have participated to the good response to IVIg, even in the presence of anti-GPIb/IX. Indeed, most of our patients (87%) received steroids in association with IVIg, as recommended by the French guidelines, which was not the case in the study of Peng *et al*. in which solely IVIg were used [8]. However, the response to IVIg was not different depending on the association or not of steroids in our study. The approach of combining IVIg with steroids is supported by the fact that it yields a higher response rate and a longer response [15]. Thus, in clinical practice, the determination of antiplatelet antibodies seems to be of little value to predict the response to IVIg in association with steroids. Importantly, in a cohort of 260 ITP patients, among which 104 had anti-GPIb/IX antibodies and received steroids, the authors observed that the presence of anti-GPIb/IX did not predict the therapeutic response, neither to steroids nor to IVIg [23].

We observed an overall response to IVIg of 75%, which is consistent with what is usually observed in ITP [12]. Despite such a high response rate, the determination of a predictive factor of response to IVIg is still of great interest to restrict their use, in an aim to decrease the medical costs and avoid adverse events. Unfortunately, no clinical predictive factor has been clearly identified yet, except for FCGR2B*232T polymorphisms, associated with a poor response to IVIg in children with ITP [28]. In our cohort of adult ITP patients, we observed that the response to IVIg was more than five times higher in patients with newly diagnosed ITP (OR = 5.5) and up to five-fold higher in the presence of anti-GPIIb/IIIa antibodies (OR = 4.8), while secondary ITP were 10 times less prone to respond to IVIg (OR = 0.1). However, it is unlikely that such criteria could be used at a patient level to determine the use or not of IVIg that still must be based on bleeding symptoms [11].

Considering the little proportion of patients that underwent splenectomy (11/69), we cannot draw definitive conclusions but the response to splenectomy did not seem to depend on platelet antibodies, with a response range between 60 and 66.6%, which is in accordance with what was observed in a large series [29].

## 5. Conclusions

We show here that when IVIg are used in combination with steroids, the presence of anti-GPIb/IX antibodies is not associated with a poor response. This is in part due to the fact that anti-GPIb/IX antibodies are rarely detected as unique antiplatelet antibodies and that they can have different effects on platelets depending on the epitope they bind. Thus, in an attempt to help managing patient treatments by determining which mechanism is involved in platelet destruction at a patient level, the evaluation of platelet sialylation could be of interest rather than determining antiplatelet antibody specificities. For clinical practice, it is worth noticing that a better response to IVIg is observed in the case of primary newly diagnosed ITP with anti-GPIIb/IIIa.

## Figures and Tables

**Table 1:** Patient characteristics according to the antigen antiplatelet antibodies.

Antiplatelet Antibodies		Anti-GPIb/IX^+^				Anti-GPIb/IX^-^				*p*
	Total	Anti-GPIIb/IIIa^+^		Anti-GPIIb/IIIa^-^	Total	Anti-GPIIb/IIIa^+^		Anti-GPIIb/IIIa^-^	Total	
		Anti-GPIa/IIa^+^	Anti-GPIa/IIa^-^	Anti-GPIa/IIa^-^		Anti-GPIa/IIa^+^	Anti-GPIa/IIa^-^	Anti-GPIa/IIa^-^		
**Clinical characteristics**										
**Population, *n***	**69**	**10**	6	1	17	2	15	35	52	
Sex ratio (Female/Male)	37/32	3/2	1/2	0	8/9	2	7/8	4/3	29/23	0.6
Age at diagnosis (years), median (IQR)	60.3 (29.8–73.6)	56.0 (30.0–70.0)	77.4 (57.0–82.1)	77.9 (NA)	68.0 (41.8–75.5)	37.9 (27.8–48.1)	65.4 (31.1–74.8)	60.3 (36.0–67.0)	59.3 (29.8–73.5)	0.6
Primitive ITP (%)	48 (69.6)	6 (60)	4 (66.67)	1 (100)	11 (64.7)	1 (50)	9 (60)	26 (66.7)	37 (71.2)	0.8
ITP course:										
- Newly diagnosed, *n* (%) - persistent, *n* (%) - chronic, *n* (%)	43 (62.3) 3 (4.3) 23 (33.3)	8 (80) 0 (0) 2 (20)	6 (100) 0 (0) 0 (0)	0 (0) 0 (0) 1 (100)	14 (82.4) 0 (0) 3 (17.65)	0 (0) 1 (50) 1 (50)	9 (60) 0 (0) 6 (40.0)	20 (57.1) 2 (5.7) 13 (37.1)	29 (55.7) 3 (5.8) 20 (38.46)	0.1 0.6 0.2
Age at IVIg initiation (years), median (IQR)	62.7 (41.8–76.4)	56.0 (33.6–69.5)	77.4 (57.0–82.1)	85.2 (NA)	66 (41.8–75.5)	40.7 (31.64–49.8)	68.1 (43.4–75.5)	62.7 (39.4–79.5)	62.5 (40.7–76.5)	1.0
**Platelet count (G/L), median (IQR)**										
At diagnosis	7 (5–18)	5.5 (5.0–8.5)	9.0 (7.0–9.8)	50 (NA)	7 (5–9)	9 (7–11)	9.5 (5.0–45.0)	7.0 (5.0–20.3)	7 (5–22)	0.2
At day 1	7 (4–14)	5.0 (3.5–8.5)	8.0 (5.5–9.8)	60 (NA)	7.0 (5–10)	7.5 (7.3–7.8)	11.0 (5.0–15.5)	7.5 (3–16.8)	8.0 (4–16.5)	0.7
At day 3	38 (11–59)	17 (10.3–33.3)	56.0 (51.5–70.3)	78 (NA)	51 (17–66.5)	25.5 (22.8–28.3)	36.0 (12.5–83.5)	40.0 (8.0–59.0)	37 (10–59)	0.6
At day 5	46.5 (18.8–91.3)	45 (31–49)	105.0 (90.5–165.5)	NA	49 (38–97)	NA	49.5 (19.8–89.0)	24 (12–66.5)	37 (13.5–79)	0.1
At day 7	43 (21–103)	33.5 (25.5–48.8)	196.0 (187.0–294.5)	NA	47 (27.5–116.5)	320 (NA)	62.0 (34.3–98.3)	36.0 (13.0–91.5)	40.5 (18.3–100.5)	0.6
At day 10	32 (14–100)	54 (20–100)	24 (NA)	NA	39 (21–88.5)	191 (NA)	77.0 (36.5–119.5)	14 (10–43)	32 (12–119.5)	0.9
**Treatments**										
Steroids (%)	60 (87.0)	9 (90)	6 (100)	1 (100)	16 (94.1)	2 (100)	12 (80)	30 (85.7)	44 (84.6)	0.4
IVIg total dose administered (g/kg), *n* (%)										NA
2 1.6 1.2 1 0.8 0.4 Not known	34 (56.7) 5 (7.2) 3 (4.3) 20 (29) 1 (1.4) 2 (2.9) 4 (5.8)	5 (50) 2 (20) 0 (0) 3 (30.0) 0 (0) 0 (0) 0 (0)	2 (33.3) 0 (0) 0 (0) 3 (50) 0 (0) 0 (0) 1 (16.6)	1 (100) 0 (0) 0 (0) 0 (0) 0 (0) 0 (0) 0 (0)	8 (47.1) 2 (11.8) 0 (0) 6 (35.3) 0 (0) 0 (0) 1 (5.9)	0 (0) 0 (0) 0 (0) 2 (100) 0 (0) 0 (0) 0 (0)	8 (53.3) 0 (0) 2 (13.3) 4 (26.7) 0 (0) 1 (6.7) 0 (0)	18 (51.4) 3 (8.6) 1 (2.9) 8 (22.9) 1 (2.9) 1 (2.9) 3 (8.6)	26 (50) 3 (5.6) 3 (5.6) 14 (26.9) 1 (1.9) 2 (3.7) 3 (5.6)	

NA = not applicable; IQR: interquartile range; GP: glycoproteins.

**Table 2:** Response to IVIg according to the antiplatelet antibody specificities.

Anti-GPIb/IX	Anti-GPIIb/IIIa	Number of Patients *n* (%)	Response to IVIg *n* (%)
+	+	16 (23.2)	15 (93.8)
+	-	1 (1.5)	0 (0)
-	+	17 (24.6)	14 (82.4)
-	-	35 (50.7)	24 (68.6)
**Total**		69	53 (76.8)

**Table 3:** Response to IVIg according to the antiplatelet antibodies in the different subgroups.

Subgroups	*n*	Anti-GPIb/IX^+^		Anti-GPIb/IX^-^		*p*
		Responders (*n*, %)	Non Responders (*n*, %)	Responders (*n*, %)	Non Responders (*n*, %)	
	**69**	15 (88.2)	2 (11.8)	38 (73.1)	14 (26.9)	0.3
Age at diagnosis ≤ 30 years	18	4 (100)	0 (0)	12 (85.7)	2 (14.3)	1.0
Age at diagnosis ≤ 60 years	33	7 (100)	0 (0)	21 (80.8)	5 (19.2)	0.6
Age at IVIg initiation ≤ 40 years	17	4 (100)	0 (0)	10 (76.9)	3 (23.1)	0.5
Age at IVIg initiation ≤ 50 years	24	5 (100)	0 (0)	16 (84.2)	3 (15.8)	1.0
Age at IVIg initiation ≤ 60 years	30	7 (100)	0 (0)	19 (82.6)	4 (17.4)	0.6
Newly diagnosed ITP	43	13 (92.9)	1 (7.1)	23 (79.3)	6 (20.7)	0.4
Persistent ITP	3	0 (0)	0 (0)	0 (0)	3 (100)	NA
Chronic ITP	23	2 (66.7)	1 (33.3)	15 (75)	5 (25)	1.0
Anti-GPIIb/IIIa antibodies	33	29 (93.8)	1 (6.3)	14 (82.4)	3 (17.7)	0.6
Anti-GPIa/IIa antibodies	12	9 (90)	1 (10)	1 (50)	1 (50)	0.3
Male	32	7 (77.8)	2 (22.2)	15 (65.2)	8 (34.8)	0.7
Female	37	8 (100)	0 (0)	23 (79.3)	6 (20.7)	0.3
Primary ITP	48	10 (90.1)	1 (9.1)	29 (78.4)	8 (21.6)	0.6
Secondary ITP	21	5 (83.3)	1 (16.7)	9 (60)	6 (40)	0.6

**Table 4:** Predictive factors of the response to IVIg.

Subgroups	Univariate Analysis		Multivariate Analysis	
	OR (CI 95%)	*p*	OR (CI 95%)	*p*
**Patients characteristics**				
Men	0.43 (0.11–1.54)	0.16	0.27 (0.06–1.28)	0.10
Secondary ITP	0.43 (0.13–1.78)	0.22	0.1 (0.02–0.64)	0.01
Age at diagnosis ≤ 30 years	2.99 (0.58–30.1)	0.21		
Age at diagnosis ≤ 60 years	2.43 (0.67–10.22)	0.16		
Age at IVIg initiation ≤ 40 years	1.55 (0.35–9.70)	0.74		
Age at IVIg initiation ≤ 50 years	2.80 (0.66–17.2)	0.15	3.0 (0.5–17.2)	0.21
Age at IVIg initiation ≤ 60 years	2.84 (0.74–13.7)	0.15		
**ITP course**				
Newly diagnosed	2.68 (0.75–10.09)	0.14	5.4 (1.2–24.7)	0.03
Persistent	NA	0.01		
Chronic	0.79 (0.22–3.10)	0.77		
**Antiplatelet antibodies**				
Anti-GPIIb/IIIa	3.56 (0.92–17.14)	0.05	4.82 (1.08–21.5)	0.04
Anti-GPIa/IIa	1.62 (0.29–16.9)	0.72		
Anti-GPIb/IX	2.73 (0.52–27.6)	0.32		

OR = Odds Ratio.

**Table 5:** Response to splenectomy according to the antiplatelet antibodies.

Anti-GPIb/IX	Anti-GPIIb/IIIa	Number of Patients *n* (%)	Response to Splenectomy *n* (%)
+	+	3 (27.3)	2 (66.6)
+	-	0 (0)	NA
-	+	5 (45.5)	3 (60)
-	-	3 (27.3)	2 (66.6)
Total		11	7 (63.4)
